# Case Report: A Mysterious Giant Thrombus in the Right Atrium in a Patient With Dilated Cardiomyopathy

**DOI:** 10.3389/fcvm.2022.954850

**Published:** 2022-06-30

**Authors:** Ping-an Lian, Xia Long, Wen-qiang Zhu, Xian-sheng Huang

**Affiliations:** ^1^Department of Cardiovascular Medicine, The Second Xiangya Hospital, Central South University, Changsha, China; ^2^Hospital Office, The Second Xiangya Hospital, Central South University, Changsha, China

**Keywords:** dilated cardiomyopathy, thrombosis, giant right atrial thrombus, cancer-associated thrombosis, lung cancer

## Abstract

An isolated right atrial thrombus is a life-threatening entity that is extremely rare in patients with dilated cardiomyopathy (DCM), which is characterized by a reduced left ventricular function and consequent left ventricular thrombosis. Here, we present the case of a mysterious isolated giant right atrial thrombus in a male patient with DCM. The presence of deep vein thrombosis prompted us to investigate for other underlying diseases for his right atrial thrombus. Interestingly, the elevation of two tumor markers indicated the likelihood of cancer-associated thrombosis. Further, the computed tomography demonstrated a spiculated mass in the lower right lung that was confirmed by an endobronchial biopsy as lung squamous cell carcinoma. Consequently, the giant thrombus in the right atrium should be attributed principally to lung squamous cell carcinoma on the background of DCM. After 3 weeks of enoxaparin, the echocardiogram indicated partial resolution of the thrombus. However, the patient suffered sudden death due to pulmonary embolism.

## Introduction

Thrombosis represents a setting of ischemia- or embolism-related pathological conditions, leading to major morbidity and mortality worldwide (1). Clinically, the thrombus is classified into the intracardiac and non-intracardiac types. Intracardiac thrombus can be identified in the left, right, or double heart. The development of intracardiac thrombus is always associated with cardiovascular disease, among which DCM is a common cardiovascular disease that easily predisposes to intracardiac thrombus (2). Typically, DCM is characterized by dilation and reduced systolic function of the left ventricle (LV), whereby it usually leads to left ventricular thrombus and systemic embolism. Here, we present a rare case of DCM with an isolated giant thrombus in the right atrium (RA). Considering the pathology of DCM, the isolated right atrial thrombus cannot be exclusively attributed to DCM, prompting us to further identify another underlying disease for this mysterious giant thrombus.

## Case Presentation

A 69-year-old male patient, a heavy smoker (200 packs/year × 40 years), was admitted to a local hospital with dyspnea on exertion 5 months prior, and then transferred to our center. Workup in the local hospital including transthoracic echocardiography (TTE) and coronary arteriography established an initial diagnosis of DCM.

**FIGURE 1:**
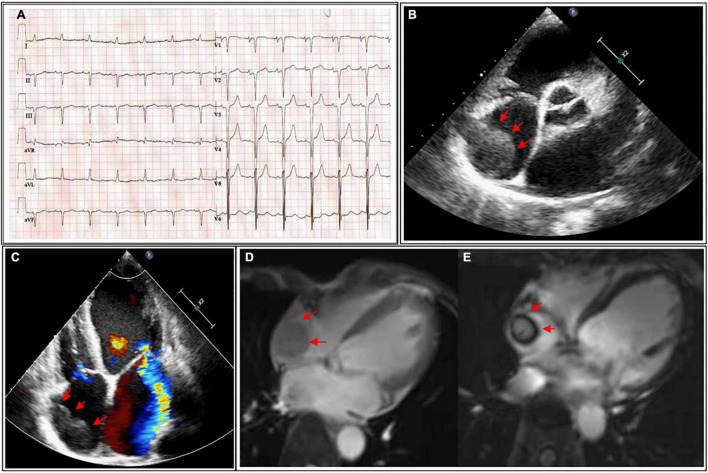
**(A)** Electrocardiogram showing poor R waves in V5 and V6, as well as QS waves in the precordial and inferior leads. **(B)** Parasternal short-axis view of transthoracic echocardiography (TTE) detecting an isolated giant mass (26 × 30 mm) in the right atrium (red arrow). **(C)** Four-chamber view with color-Doppler of TTE showing a giant mass in the right atrium (red arrow) and severe mitral regurgitation. **(D)** T1-weighted imaging of cardiac magnetic resonance imaging (CMR) indicating the mass in the right atrium (red arrow) with medium-to-high signal intensity. **(E)** Delayed enhancement imaging of CMR identifying a thrombotic nature mass (red arrow). TTE, transthoracic echocardiography; CMR, cardiac magnetic resonance imaging.

A physical examination was noteworthy for jugular venous pulsations and a grade 3/6 systolic murmur auscultated at the mitral area. His laboratory examinations showed N-terminal prohormone BNP of 1930.84 pg/ml (reference, 0–125 pg/ml), platelet count of 58 × 10^9^ cells/μL (reference, 125–350 × 10^9^ cells/L), prothrombin time of 21.4 s (reference, 10–14 s), activated partial thromboplastin time of 49.7 s (reference, 23–35 s), fibrinogen level of 1.06 g/L (reference, 2.0–4.0 g/L) and D-dimer of 14.73 μg/mL (reference, <0.55 μg/ml). An electrocardiogram demonstrated poor R waves in V5 and V6 as well as QS waves in the precordial and inferior leads (Figure 1A). The TTE indicated an enlarged left heart (LV, 61 mm; left atrium, 42 mm), diffuse weakening of LV wall motion, and decreased left heart function (ejection fraction, 24%), consistent with the characteristics of DCM. Of note, the TTE depicted a homogeneous hypoechoic, hypermobile giant mass (26 × 30 mm) in the right atrium attached to the anterior atrial wall *via* a stalk (Figures 1B,C). His ultrasound of the lower extremities detected multiple venous thromboses. This occurred in the absence of a recent myocardial infarction. No atrial septal defect or patent foramen ovale was noted. To identify whether the RA mass was a thrombus or a cardiac tumor, cardiac magnetic resonance imaging (CMR) was performed. Cine imaging of CMR found a highly mobile, homogeneous, and globe-shaped mass measuring 32 mm × 24 mm and filling approximately 60% of the RA. T1- and T2-weighted perfusion images indicated hypointense characteristics of this RA mass, and delayed enhancement imaging showed a homogeneous gray mass with a dark rim and no uptake of contrast, suggesting a mass of a thrombotic nature, rather than a myxoma or malignant tumor (Figures 1D,E).

**FIGURE 2:**
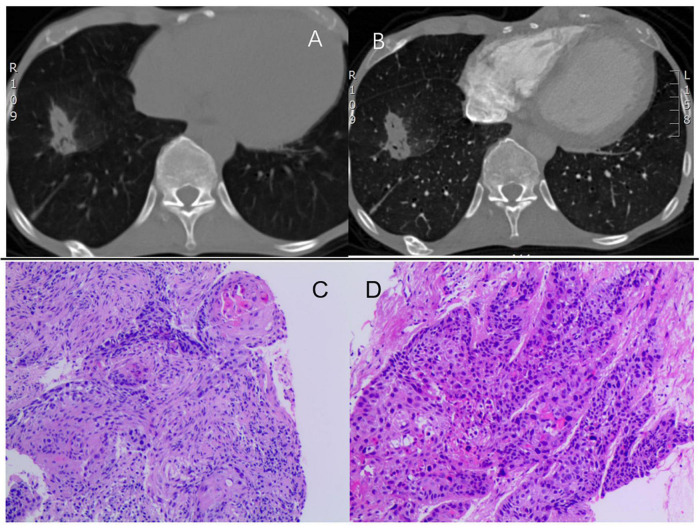
**(A,B)** Computed tomography showing a spiculated mass (35 × 20 mm) in the lower right lung lobe. **(C,D)** H&E staining identifying lung squamous cell carcinoma with no keratinization but well-defined nests. H&E, hematoxylin and eosin staining.

However, an isolated right atrial thrombus is rare in patients with DCM. Additionally, the presence of deep vein thrombosis (DVT) and hypercoagulable state indicate another systemic disease. Further examinations were conducted to investigate the etiology of the isolated right atrial thrombus. Results of autoimmune or connective tissue diseases were negative. Notably, two tumor markers were elevated, including carbohydrate antigen 19-9 and cancer antigen 125. Computed tomography scans demonstrated a spiculated mass lesion measuring 35 × 20 mm in the lower right lung lobe (Figures 2A,B), which was further confirmed by an endobronchial biopsy as lung squamous cell carcinoma (Figures 2C,D). Piecing all the diagnostic clues together, the isolated right atrial thrombus was primarily attributed to lung squamous cell carcinoma, indicating the possible existence of cancer-associated thrombosis in this patient. Considering his heart failure and lung cancer, surgical removal of the thrombus was declined by the patient. Instead, enoxaparin (1.5 mg/kg subcutaneously daily), heart failure medication (furosemide 20 mg once daily, spironolactone 20 mg once daily, Betaloc ZOK 47.5 mg once daily, sacubitril/valsartan 50 mg twice daily) were recommended. The patient was also evaluated by the thoracic oncology group and was ultimately diagnosed with borderline resectable lung squamous cell carcinoma. Neoadjuvant therapy was prudently recommended for his lung squamous cell carcinoma after improvement of cardiac function, with the hopes of surgical resection in the future.

Written informed consent was obtained from the patient for the publication of any potentially identifiable images or data included in this article.

## Discussion

We have reported a rare case of DCM with an isolated giant right atrial thrombus. Combining CT imaging and endobronchial biopsy, the diagnosis of lung squamous cell carcinoma was established, which is regarded as the major underlying disease for the mysterious giant thrombus. To our knowledge, lung cancer with an isolated giant right heart thrombus has only been reported in a few literatures (3, 4), and it is the first time to be reported in a patient with DCM.

Thrombosis is a serious complication of DCM, for severe adverse outcomes. Notably, DCM is always associated with left ventricular thrombosis and systemic thromboembolic events including stroke, myocardial infarction, and peripheral arterial thromboembolism, leading to increased morbidity and mortality in subjects with DCM (2). Pathologically, DCM is characterized by ventricular dilatation and dysfunction. More importantly, ventricle dilatation in DCM starts from left ventricular (LV) remodeling and fibrosis, resulting in LV myocardial damage and reduced systolic function (5, 6). As a result, a series of prothrombogenic factors are implicated, including blood stagnation, local myocardial injury, and reduced wall motion, which are followed by LV thrombosis and systemic embolism (7). Consequently, DCM-associated thrombosis is most common in the LV rather than the right heart.

Of note, although the cardiac magnetic resonance imaging (CMR) imaging demonstrated striking wall-motion abnormality and fibrotic scar in the LV, no thrombi were detected in the LV. Instead, an isolated giant right atrial thrombus was showed in this patient. Therefore, it prompted us to investigate another non-cardiac disease underlying the isolated RA thrombus, because of (1) the lack of evidence of LV thrombus and systemic embolism in this patient; (2) the isolated RA thrombus being pathologically unexplained by DCM; (3) the presence of DVT and coagulation disorders suggesting a prothrombotic state and another systemic disease. Ultimately, CT scanning and endobronchial biopsy confirmed the diagnosis of lung squamous cell carcinoma. Pathologically, the formation of RA thrombus should be primarily attributed to lung cancer on the background of DCM in this case.

Thromboembolic events are listed as common and severe complications that contribute to the second cause of death in patients with cancer (8). Risk factors of cancer-associated thrombosis include tumor type, surgery, chemotherapy, and central venous catheterization (9). Regarding its pathological and clinical nature, cancer-associated thrombus accounts for approximately 20% of all incidents of venous thromboembolism (VTE) (10). A recent cohort study has shown that 9.23% of patients with lung cancer had a diagnosis of VTE (11). The incidence of thrombosis is as high as 13.6% throughout non-small cell lung cancer cases (11) and the lung adenocarcinoma accounts three-fold higher than squamous cell carcinoma, which may be attributed to increased mucin (12). The pathogenesis of cancer-associated thrombosis is multifactorial and involves all aspects of Virchow’s triad. Additionally, the prothrombotic state of malignant tumors is also driven by cancer-specific pathways, including tumor-related leukocytosis and release of tissue factor, podoplanin, as well as plasminogen activator inhibitor 1 (13). Right heart thrombi were associated with abruption of DVT in prior cases, which accidentally lodged in the right heart while in transport to the lungs (3, 4).

Overall, three patterns of right heart thrombi (A, B, and C) have been identified by the European Working Group on Echocardiography, suggesting respective etiologies (14). Type A right heart thrombus is morphologically serpiginous or worm-shaped and extremely mobile, with variable shapes, which is associated with abruption of DVT in patients with thrombophilic states, while cardiac abnormalities are rare in these patients (14–17). Type B is less mobile without a specific shape that is believed to develop within the right heart with a lower incidence of VTE and a high incidence of cardiac predisposing factors. Assuming intermediate characteristics, type C is highly mobile and shares a similar appearance to a myxoma but is rarely described in the current literature. The right atrial thrombus in our patient should be categorized as type C because it is not worm-shaped (unlike type A) but highly mobile (unlike type B). Although most of cancer-associated thrombi in the right heart are categorized as Type A, but it is also indicated that right heart thrombi will develop a myxoma-alike shape due to its folding and agglutination *per se* (14).

As to therapeutic strategies, a type A right heart thrombus is recommended for surgical embolectomy and thrombolytics because of the high incidence of pulmonary embolism, while type B is treated with anticoagulation due to its benign course (14). There is no recommendation for the optimal management of type C in the literature. Thrombectomy was performed in prior cases, whereas its risk-benefit profile still remains unknown (18). For our patient, surgical removal of the thrombus was not acceptable due to his heart failure and lung cancer. As it is considered ineffective and unsafe for giant thrombi (19), thrombolytic treatment was also not recommended (19). Instead, low molecular weight heparin (LMWH) was deemed reliable for this patient because LMWHs are recommended as the standard treatment for cancer-associated thrombosis (20, 21). After 3 weeks of enoxaparin, the patient was followed up at the local hospital and his echocardiogram showed partial resolution of the thrombus in the left atrium. Unfortunately, the patient suffered sudden death due to acute pulmonary embolism.

## Conclusion

We reported a rare case that an isolated giant right atrial thrombus in a patient with DCM. The patient has been finally diagnosed with lung squamous cell carcinoma, which was regarded as the major cause for the mysterious giant thrombus in the RA. According to the characteristics of the thrombus and the clinical profile of this patient, the isolated right atrial thrombus was categorized as a type C right heart thrombus. The patient’s diagnostic course emphasizes the necessity for clinicians to investigate another underlying non-cardiac disease for unexplained intracardiac thrombosis.

## Data Availability Statement

The raw data supporting the conclusions of this article will be made available by the authors, without undue reservation.

## Ethics Statement

Written informed consent was obtained from the individual(s) for the publication of any potentially identifiable images or data included in this article.

## Author Contributions

P-AL, XL, and W-QZ collected and analyzed the patient data. P-AL and XL wrote the manuscript. X-SH provided the supervision. All authors approved the final version.

## Conflict of Interest

The authors declare that the research was conducted in the absence of any commercial or financial relationships that could be construed as a potential conflict of interest.

## Publisher’s Note

All claims expressed in this article are solely those of the authors and do not necessarily represent those of their affiliated organizations, or those of the publisher, the editors and the reviewers. Any product that may be evaluated in this article, or claim that may be made by its manufacturer, is not guaranteed or endorsed by the publisher.
